# Can FinTech Development Curb Agricultural Nonpoint Source Pollution?

**DOI:** 10.3390/ijerph16224340

**Published:** 2019-11-07

**Authors:** Song Jiang, Shuang Qiu, Hong Zhou, Meilan Chen

**Affiliations:** 1 School of Economics and Finance, Chongqing University of Technology, Chongqing 400054, China; jiangsong@cqut.edu.cn (S.J.); holiday@2015.cqut.edu.cn (S.Q.); 2 Chongqing Research Center of Labor Economics and Human Resources, Chongqing 400054, China; 3 Chongqing 208 Survey and Design Institute, Chongqing 400700, China; zhouhong4737@163.com; 4 International Business School, Guangdong University of Finance and Economics, 1 Xuehai Road, Foshan 528100, Guangdong, China

**Keywords:** FinTech, agricultural NPS pollution, structural effect, threshold model, China

## Abstract

The green development of FinTech empowerment has become a compelling theme in economic development. In this study, based on the weighted least squares (WLS) and threshold regression methods of cross-sectional data, we empirically examine the impact of FinTech development on agricultural nonpoint source (NPS) pollution, a major cause of impaired surface water quality. Our results show that there is an inverted “U” shape relationship between the development of FinTech and agricultural NPS pollution. That is, after crossing a “threshold value”, the level of FinTech development can curb agricultural NPS pollution. At the structural level, the availability of FinTech services, the FinTech infrastructure, and the agricultural NPS pollution also have an inverted “U” shape relationship. At the threshold effect, in the developing stage of an agricultural economy, the overall level of FinTech development, the use of FinTech services, the availability of FinTech services, and the FinTech infrastructure have an inverted “U” shape relationship with agricultural NPS pollution. On the other hand, in the developed stage of an agricultural economy, the impact of FinTech development and its structure on agricultural NPS pollution is insignificant. Hence, we can conclude that FinTech development can help reduce agricultural NPS pollution in under-developed regions. However, due to the fact that a “U” shape relationship always exists between FinTech service quality and agricultural NPS pollution, the quality of FinTech service should be the main focus to reduce agricultural NPS pollution more effectively.

## 1. Introduction

Nonpoint source (NPS) pollution, such as agricultural runoff of nutrients and sediment, is the chief cause of impaired surface water quality today [1,2,3]. A defining characteristic of NPS pollution is its ubiquitous nature [4]. Even though its forms vary throughout the world, NPS pollution is a major global environmental issue. As a matter of fact, due to NPS pollution, hundreds of millions of people suffer from disease, billions of dollars of economic development investments are lost, and trillions of dollars of environmental remediation needs are being accumulated for future generations to address [5]. Controlling agricultural NPS pollution is hence a key to agricultural transformation and green development in many developing countries. As a result, the governments in many countries have begun to pay attention to the environmental issues in agricultural production and modernization. Agricultural NPS pollution prevention and control has gone from unimportant to critical parts of their environmental management framework. However, judging from the early empirical data, agricultural NPS pollution control so far has been unsatisfactory, trapped into a “policy failure” dilemma in developing countries: capital investment is increasing year by year, but the agricultural NPS pollution is getting worse. As it stands right now, agricultural NPS pollution has become the cradle of pollution, contaminating the atmospheric environment and water resource. Thus, innovating the agricultural NPS pollution control has become an urgent problem to address to achieve high-quality economic development.

Special attention has been paid to develop pollution control policies based on economic incentives [6]. However, the dispersibility, concealment and hysteresis characteristics of such pollution have contributed to the ineffectiveness of measures and policies relying on legal and administrative means. Agricultural NPS pollution control must continuously improve with the appropriate policy “toolbox”. After the government-dominated administrative fiscal policy gets stuck in a “failure” dilemma, the market-led and multi-agent “self-organizing” financial instruments must be implemented to play a more prominent role. This is the general empirical finding on agricultural NPS pollution control in developed countries as well. In the United States, for example, guided by the NPS pollution control action plan, the federal government has set up a $50 billion clean water fund as a “seed funding” to support farmers and agriculture-related enterprises and entities to minimize agricultural NPS pollution by the means of interest-free or low-interest loans [7]. Compared with administrative means, market-based financial means can enhance the initiatives of agricultural production and management entities to engage in agricultural NPS pollution control by providing direct financing and funding platform. This can lead to a new pattern of coordinated governance. In short, financial means can improve the efficiency and effectiveness of agricultural NPS pollution control.

In developing countries, agricultural production and operations entities such as farmers and agribusinesses have been in a “financial exclusion” state chronically. Market-oriented financial instruments have been in a long “hibernating” state, and the governance effects have not been fully stimulated and thoroughly exerted. Fortunately, with the implementation of information technology in agriculture, the situation is quietly improving. The “catalyst” of change is the financial technology (FinTech) which is in full swing. Globally, investment in FinTech (mainly loans) grew from $4.05 billion in 2013 to $12.21 billion in 2014 [8]. And the amount reached $57.9 billion in the first half of 2018. As a combination of finance and technology, FinTech utilizes innovative technologies such as big data, cloud computing, artificial intelligence, and blockchain to innovate the products and services provided by traditional financial industries, to improve efficiency and reduce operating costs, and to resolve the long-term problem of “financial exclusion” which has plagued those developing countries for a long time. Besides, the costs associated with accessing financial services are lower [9]. In other words, FinTech provides new access to financial services for farmers, agriculture-related enterprises and other “long tail” crowd. 

At the same time, FinTech can bring about new challenges and risks. Although a large amount of investment has entered the market for the green development of agriculture, the results have been unsatisfactory. The same is true for FinTech in terms of its green development effect and its inhibiting effect of agricultural NPS pollution. Therefore, how does the rapidly emerging FinTech affect agricultural NPS pollution? What are the characteristics of its effect? Our study here makes an important contribution to both theory and practice by aiming to answer these questions. This is especially true because FinTech is the forefront of world financial innovation and development. In developing countries, FinTech has achieved revolutionary and explosive development. The role of FinTech has begun to emerge in financing small and micro enterprises and low-income groups for green development. For example, platforms such as “Ant Forest” on Alipay have shown unprecedented development potential in green development. So far, over 56 million “ant trees” have been planted by players across the country with promising results. For example, the Saihanba Plateau in China, once a dusty wasteland, has become the world’s largest manmade forest [10]. Globally, 30–50% of the Earth’s surface has been affected by NPS pollution, and a large part of NPS pollution is agricultural NPS pollution [4]. Thus, our analysis and results in this study can assist with agricultural NPS pollution control, especially in the emerging market countries with more pronounced “financial repression” issues.

The main contributions of this research are as follows: First, with the development of green finance, there appears to be an urgent need to study the impact of financial development on agricultural NPS pollution from the perspective of financial means. Hence, this study can enrich the “toolbox” for agricultural NPS pollution control. Second, FinTech has been at the forefront of financial development. However, there appears to be a research gap on linking FinTech with agricultural NPS pollution control. Our study fills this research gap. 

The rest of this paper is organized as follows: Section 2 presents the literature review; Section 3 details our research methods; Section 4 explains the variables and data used in this research; Section 5 presents our research results followed by discussions; Finally, Section 6 summarizes our research findings and suggests future research directions.

## 2. Literature Review

### 2.1. Research on FinTech

FinTech is a new business model that combines financial services innovation with the latest Internet technologies [11]. Since FinTech is still a relatively new industry, its meaning is not well defined. However, scholars generally agree that FinTech is a technical means that applies science and technology to the financial industry, serves the general public, reduces industry costs, and improves industry efficiency. It extensively affects financial payment, financing, loans, investment, financial services, and currency operations [12]. FinTech has become the driving force of financial demand discovery, financial product and service innovation, and social wealth creation. It can be said that FinTech is the commanding height of the future financial industry, playing a strategic and decisive role in the next round of global financial development with huge market potential. According to recent statistics, 38% of the world’s population has no official bank account, and another 40% cannot obtain adequate bank services. This indicates an enormous market for FinTech development [13].

FinTech research has attracted widespread interest among scholars. Lee and Teo [8] believe that although FinTech is at its initial stage of development, it will define and reshape the future of the financial industry. At the same time, FinTech enhances financial inclusion and accelerates “disintermediation”, and it provides a new level for the development of the real economy, especially in areas with poor economic performance, where FinTech has a greater scope to function. On the other hand, some scholars advocate that financial capital can also enter a new “risk boundary” within the current institution and policy framework [14]. The entire financial service industry has been fundamentally disrupted [15]. Fenwick et al. [16] explored the influence of FinTech on household credit scoring. On the one hand, they believed that FinTech companies could use big data to improve the scoring accuracy of algorithms, reduce the cost and improve the convenience of credit access. Nevertheless, in their view, the algorithms also have a potential “dark side” of illegal statistical discrimination. They believed the FinTech use, especially big data and algorithm scoring, could increase the degree of loan discrimination. For example, they find that African American and Hispanic borrowers had a loan rejection rate of 5% higher. Legal risk is also a major problem in the development of FinTech [17]. Jagtiani and Lemieux [13] argued that FinTech could place banks in an unfair competitive environment because FinTech companies were subject to different regulatory conditions. For example, The Federal Deposit Insurance Corporation (FDIC) and the Consumer Financial Protection Bureau (CFPB) are concerned about the consumer credit access conditions and credit privacy offered by FinTech companies. That’s why the new developments in financial high-tech dominated by start-ups have challenged both regulators and market participants, especially in balancing the potential benefits of innovation and the risks. 

Affected by disruptive innovation and the balance of financial stability and innovation, FinTech supervision should adhere to the basic principles of containing systemic risks and protecting consumer interest to promote innovation and improve the inclusiveness of digital finance to appropriately handle the balance between FinTech innovation and supervision [18]. Only in this way can the positive effects of financial science and technology be achieved. In this regard, this paper reveals the impact of the development of financial science and technology on the control of agricultural NPS pollution, which can help design innovate regulatory methods to improve the environment.

### 2.2. Research on Agricultural NPS Pollution Control

The theory of agricultural NPS pollution control has been developed for a long time [19]. This research began in developed countries such as the United States and the United Kingdom in the 1960s. There are two types of economic policy: “Pigou means” and “Coase means”. “Pigou means” focus on vertical government intervention, and “Coase means” emphasize horizontal adjustment of market mechanisms [20].

In the “Pigou means”, taxes, financial subsidies, and administrative interventions are the most widely used tools. In terms of tax policy, Griffin and Bromley [21] took the lead in advocating for tax on agricultural means of production, such as pesticides and fertilizers, to control agricultural NPS pollution. Shortle and Dunn [22] also affirmed this “input tax”. In a follow-up study, Segerson [23] established a mechanism including “fixed penalty + overall taxation” to tax polluters. Under this mechanism, taxation policies are more efficient, but when there is informational asymmetry, polluters can obtain higher profits through “collusion” [24]. Consequently, some scholars have suggested that with incomplete information, the key to agricultural NPS pollution control was to motivate farmers [25]. To this end, Ribaudo [26] and Griesinger et al. [27] advocated for the use of financial subsidies to guide farmers to employ “green technology”. In terms of administrative intervention, Egan and Mahoney [28] advocated strengthening judicial, legislative and regulatory procedures for the agricultural NPS pollution control. As a whole, “Pigou means” requires a much more active government participation and higher management cost [29].

In the “Coase means”, tools such as sewage charges and carbon emission permit trading are widely used. They are generally considered to be effective or cost-effective [30], which can encourage polluters to adopt new technologies and reduce the marginal benefits of violations [31]. However, whether they can achieve the policy objective depends on the initial allocation quantity and distribution. For example, Tanaka [32] constructed a multi-sector emission trading model that included oligopoly and perfect competition industries. A finding is that increasing the initial allocation of emissions rights in the oligopoly industry would increase its output levels. Moreover, raising the initial allocation of “cleaning” companies would lead to a decline in output and license prices. However, both environmental regulators and companies tend to distribute licenses freely because it is possible to build “entry barriers” and bring rental income [33]. In fact, it also increases the cost of governance and restricts the participation from other market players, leading to inefficient governance. Therefore, to improve the efficiency of governance, an auction mechanism should be established [34]. In this way, the distribution of licenses depends on the licensed market price, market demand, and the use of “cleaning technology”, thereby reducing distribution costs and improving governance efficiency.

As a whole, the “Pigou means” has great limitations both on the subjective level and tool level. Agricultural NPS pollution control still needs to be explored from the level of “Coase means”. Financial instruments, especially the newly developed FinTech, are very important in the “Coase Means”.

Based on the literature review so far, more research is still needed in the following aspects. The control methods and tools for agricultural NPS pollution still rely on administrative means, so the economic policy toolbox needs to be further expanded. As an important part of economic policies, the financial policy should play an important role in agricultural NPS pollution control. Therefore, it is important to study the impact of financial development on agricultural NPS pollution control. What’s more, most developing countries have always faced the financial repression problems, but with the help of FinTech, these problems can be alleviated. At present, with the rapid development of data technology represented by distributed technology, interconnect technology, artificial intelligence, the FinTech industry is rapidly emerging. It is necessary and urgent to reveal and evaluate its impact on agricultural NPS pollution. Research in this field is still very rare. On the one hand, our research results can improve the market-oriented toolbox for agricultural NPS pollution control. On the other hand, our research can also diagnose the current “green development effect” of FinTech, identifying existing problems, proposing appropriate solutions, and helping to achieve sustainable development.

## 3. Research Methods

### 3.1. The Theoretical Model

Research on environmental economics has revealed the main factors affecting environmental pollution. In early theoretical research, Ehrlich and Holdren [35] argued that population growth would have a disproportionately negative impact on the environment, and issues such as population size and growth, resource utilization and environmental degradation must be considered globally. The total negative impact of a society on the environment (I) can be simply expressed as:

(1) I=P×F

In Equation (1), P is population size, and F is a function to measure per capita influence. In Ehrlich and Holdren’s view, this simple relationship is more complex than it seems. When technology stays the same, F will increase with per capita consumption. However, in some cases, such as introducing environmentally friendly technologies in the process of providing a constant consumption level, F may also be reduced. Since per capita impact itself is a function of population size, Equation (1) can be further expressed as:

(2) I=P×F(P)

Equation (2) implies that as the population increases, the per capita negative impact will grow faster. Of course, whether F(P) is an increasing or decreasing function of P mainly depends on the income and the level of scale economy. In some industrialized countries, most scale operations have been developed. Therefore, the reduction of income is the most significant factor for negative impact. This is also tenable in agricultural production. In the case of declining returns, the food need of a growing population is generally met by sacrificing the environment. In agricultural production, farmers may increasingly use fertilizers, pesticides and energy to enhance food production. However, this will also lead to ecological overload, environmental damage, and increased agricultural NPS pollution. In summary, the factors affecting environmental pollution constructed by Ehrlich and Holdren [35] can be summarized as three factors: population size, technical level, and affluence. Therefore, Equation (2) can be rewritten as the following “IPAT” model:

(3) I=P×A×T

where *A* is the affluence level and *T* is the technical level. Since this model only assumes a priori proportional relationship among various factors, it does not allow for hypothesis testing. Consequently, based on the theoretical framework of Ehrlich and Holdren [35], empirical models have been established in the literature. In a representative work, York et al. [36] built the following “STIRPAT” model:

(4) $$I=aP_{i}^{b}\times A_{i}^{c}\times T_{i}^{d}e_{i}$$

In Equation (4), I represents a country or a region, e is a random error term, and a,b,c,d are parameters to be estimated. Note that Equation (4) can be converted into a linear equation by logarithmic treatment. Therefore, the estimated parameters can be regarded as the elasticity of the population size, affluence level, and technical level on environmental pollution. It should be noted that, as this study focuses on the impact of FinTech development on agricultural NPS pollution, the technical level in the model is mainly limited to the FinTech level. Therefore, the effect of FinTech on environmental pollution essentially reflects the compound impact of financial development and technological level on agricultural NPS pollution. Compared with the “STIRPAT” model framework, it is more valuable and practical than just listing financial development as the single variable to evaluate the environmental effects of financial development [37]. At the same time, the multicollinearity between technological progress and financial development can be well solved by incorporating the level of FinTech development into the category of technological progress.

### 3.2. The Empirical Model

Based on the theoretical model, the empirical model used in this study is further constructed. First, taking the natural logarithm of both sides of Equation (4), we can get:

(5) lnI=lna+blnp+clnA+dlnT

By estimating model (5), the direction and the elasticity of each variable can be obtained. As the data type used in this study is cross-sectional, it is prone to heteroscedasticity. Under such conditions, the estimation results of ordinary least squares (OLS) are biased and inconsistent. To overcome the heteroscedasticity, the most common processing method is to re-estimate the model by weighted least squares (WLS), where the original model is weighted to generate a new model without heteroscedasticity, and then using the ordinary least squares method to estimate its parameters. If the weight is assumed to be $w_{i}=\frac{1}{\sigma_{i}}$, the corresponding matrix form is as follows:

(6) $$W=\left(\begin{array}{cccc} w_{1} & 0 & \cdots & 0\\ 0 & w_{2} & \cdots & 0\\ \cdots & \cdots & \cdots & \cdots \\ 0 & 0 & \cdots & w_{N} \end{array}\right)$$

After using the weighted least squares estimation, this paper follows the methods of Godfrey [38], Breusch [39], Harvey [40], Glejser [41], Bera [42], and White [43] to further test the model heteroscedasticity problem. As the variables are nonlinear, in the latest development of the “STIRPAT” model, some studies, such as York et al. [36], further modified model (5) and introduced the secondary item of affluence to reflect the dynamic change of people’s income. Then model (5) can be further transformed into:

(7) $$lnI=lna+blnP+\left[c_{1}lnA+c_{2}{\left(lnA\right)}^{2}\right]+dlnT$$

Compared with the nonlinear model and research focus of York et al. [36], this study aims to reveal the impact of FinTech on agricultural NPS pollution and its dynamics. Therefore, in this study, ${\left(\mathrm{lnT}\right)}^{2}$ is introduced to reflect the dynamic changes and the phased characteristics of the FinTech’s effect. Then the model can be transformed into:

(8) $$lnI=lna+blnP+clnA+\left[d_{1}lnT+d_{2}{\left(lnT\right)}^{2}\right]$$

The weighted least squares method can be used to estimate the model (8). Generally speaking, at different stages of agricultural economic development, the impact of FinTech on agricultural NPS pollution may be significantly different. At present, the agricultural economy has entered a new stage of structural adjustment in many countries. To reveal the characteristics of this stage and the incoming transformation, Equation (8) has been rewritten into a threshold regression model, following Hansen [44]. To this end, the level of agricultural economic development (q) is set as a threshold variable, and its corresponding threshold is $q^{*}$. Then the model (8) can be transformed into:

(9) $$\{\begin{array}{c} lnI=lna_{1}+b_{1}lnP+c_{1}lnA+\left[d_{1}^{1}lnT+d_{1}^{2}{\left(lnT\right)}^{2}\right],q\geq q^{*}\\ lnI=lna_{2}+b_{2}lnP+c_{2}lnA+\left[d_{2}^{1}lnT+d_{2}^{2}{\left(lnT\right)}^{2}\right],q<q^{*} \end{array}$$

After the model is set up, the next critical step is to determine whether the threshold of agricultural economic development $q^{*}$ exists. Following Hansen [44], ${\hat{q}}^{*}$ is the regression value of $q^{*}$, which should be the value from the minimum squared residual regression. It can be expressed as:

(10) $${\hat{q}}^{*}=\underset{q*}{\underbrace{argmin}}S_{n}\left(q^{*}\right)$$

Therefore, after the first threshold is determined, the next key is to check the number of threshold values to illustrate whether there are significant differences in the classification groups and the estimated parameters. Therefore, the original hypothesis is “there is no threshold effect” and is expressed as: $H_{0}:\;b_{1}=b_{2};c_{1}=c_{2};d_{1}^{1}=d_{2}^{1};d_{1}^{2}=d_{2}^{2}$. Following Hansen [44], the hypothesis was tested primarily by constructing LM statistics:

(11) $$F=n\frac{S_{0}-S_{n}\left({\hat{q}}^{*}\right)}{S_{n}\left({\hat{q}}^{*}\right)}$$

In Equation (11), $S_{0}$ is the residual sum of squares under the null hypothesis, $\mathrm{and}\;S_{n}\left({\hat{q}}^{*}\right)$ is the residual sum of squares under the threshold effect. However, the statistical test of Equation (11) can lead to the traditional test statistics whose large sample distribution is not “chi-square distribution”, but “non-standard and non-similar” distribution affected by interference parameters. To overcome this problem, Hansen [44] transformed the large sample distribution function of the statistic itself to obtain the asymptotic p value of the large sample. Under the null hypothesis, the large sample distribution of the p value statistic is uniform and can be calculated by bootstrap. The basic idea of this method is to simulate a set of dependent variable sequences and make them satisfy N (0:${\hat{e}}^{2}$), where ${\hat{e}}^{2}$ is the residual term of Equation (9). For each self-sampled sample, a simulated LM statistic can be calculated and the process will be repeated 1000 times. 

When giving the threshold effect of a variable, the confidence interval of its threshold value needs to be further determined. That is, the null hypothesis is: $H_{0}:\;q^{*}=q_{0}^{*}$. The likelihood ratio statistic can be expressed as:

(12) $$LR_{n}\left(q_{0}^{*}\right)=n\frac{S_{n}-S_{n}\left({\hat{q}}^{*}\right)}{S_{n}\left({\hat{q}}^{*}\right)}$$

In Equation (12), $LR_{1}$ is also a non-standard normal distribution. Hansen [44] calculates its confidence interval and finds that is when the significant level is γ and $LR_{1}\left(q_{0}^{*}\right)\leq c\left(\gamma \right)=-2ln\left[1-\sqrt{\left(1-\gamma \right)}\right]$, the null hypothesis cannot be rejected. Moreover, at the 95% confidence level, c(γ)=7.35.

## 4. Variables and Data

### 4.1. Variable Description

#### 4.1.1. Dependent Variables

Agricultural NPS pollution (I) is a dependent variable in the empirical model. Agricultural pollution is mostly NPS pollution, with characteristics such as dispersibility, concealment and hysteresis. It is mainly composed of soil sediment particles, nitrogen and phosphorus nutrients, pesticides, and various atmospheric particulates. Through surface runoff, soil erosion and farmland drainage, agricultural pollution enters the water, soil or atmospheric environment. Thus, the chemical fertilizer (CF) used in agricultural production, pesticide (PE), agricultural plastic film (PL) and the diesel oil (DF) used in the process of agricultural machinery are the main sources of pollution in the agricultural production. When measuring the agricultural NPS pollution, scholars mostly weigh the main pollution sources in agricultural production. Common methods include analytic hierarchy process and principal component analysis. However, the analytic hierarchy process mainly relies on the scoring from experts so it is more subjective. The principal component analysis is a type of “dimension reduction” techniques, but the weight of each indicator cannot be accurately reflected. At the same time, with a large number of indicators, problems of information crossover and overlap among variables can be severe. Hence, this study adopts a simple and transparent equal-weight method of Human Development Index (HDI) prepared by the United Nations Development Programme (UNDP) to assign values of the major pollution sources in agricultural production [45]. Thus, the weights of fertilizer, pesticide, agricultural mulch film and diesel are all set at 0.25, then the level of agricultural NPS pollution can be expressed as:

(13) I=0.25×CF+0.25×PE+0.25×PL+0.25×DF

#### 4.1.2. Independent Variables

FinTech (T): Because of its unique market, China’s FinTech development has attracted worldwide attention. In China, there is a FinTech development index system which was jointly compiled by the National Finance and Development Laboratory, the Institute of Finance of the Chinese Academy of Social Sciences and the Investment and Financing Research Center of the Chinese Academy of Social Science. Based on the four aspects of FinTech services, the availability of FinTech services, the FinTech infrastructure and the quality of FinTech services, the index system was built and the FinTech inclusive financial index was synthesized. The published indexes mainly consist of five components: FinTech inclusive financial index ($T_{F}$), FinTech service use index ($T_{U}$), FinTech service availability index ($T_{G}$), FinTech infrastructure index ($T_{I}$), and FinTech service quality index ($T_{Q}$). Therefore, this study is carried out from the overall and structural levels when conducting the test. The test of the overall level of FinTech mainly uses $T_{F}$. The test of the structural level of FinTech development mainly utilizes $T_{U}$, $T_{G}$, $T_{I}$ and $T_{Q}$.

Population (P): The expansion of rural population will form a strong consumption demand for agricultural products like grain and agricultural resources. This will increase the total amount of domestic sewage and garbage, aggravating the agricultural NPS pollution. At the same time, in order to meet the growing demand for food, agricultural producers will inevitably increase the input of agricultural production factors such as fertilizers and pesticides to improve food production, which will also intensify NPS pollution. Therefore, the size of rural population is a very important factor affecting agricultural NPS pollution. In this paper, rural population is used to represent population size.

Affluence (A): Affluence is also an important indicator of agricultural NPS pollution. This can be confirmed from the Environmental Kuznets Curve (EKC) [46]. When a country’s economic development level is low, the degree of environmental pollution is relatively low. As per capita income increases, the level of environmental pollution rises. When economic development reaches certain critical point or “inflection point”, with the further improvement in per capita income, environmental pollution begins to decrease, and environmental quality gradually improves. Hence, we can hypothesize an inverted “U” type relationship between affluence level and agricultural NPS pollution. To best it, this paper uses the disposable income of rural residents.

#### 4.1.3. Threshold Variables

Agricultural economic level (q): Generally speaking, agricultural NPS pollution and its level of governance are closely related to the rate of economic growth and the stage of economic development. At different levels of economic development, a government’s emphasis on environmental issues and interventions can also be different. Therefore, in addition to factors mentioned above, the agricultural economic level must be taken into account. Also, the emergence of FinTech, population size and affluence are closely related to the overall scale, development level and growth rate of the agricultural economy. In other words, at different stages of the agricultural economy, indicators such as agricultural NPS pollution, FinTech, population size and affluence have different characteristics and manifestations. To reflect these phased characteristics, this study sets the level of the agricultural economy as a threshold variable, revealing the differences and characteristics of the effects at different economic development stages. As for the specific measurement, it is expressed by the added value of agriculture, forestry, animal husbandry, and fishery.

### 4.2. Data Description and Processing

The data used in this study are cross-section data of 31 provinces and regions in mainland China in 2017. The data of fertilizer, pesticide, agricultural film and diesel used to measure agricultural NPS pollution levels are from China Rural Statistical Yearbook; The indicator data of FinTech inclusive financial index, FinTech service use index, FinTech service availability index, FinTech infrastructure index and the FinTech service quality index mainly come from the China Internet Network Development Statistics Report released by China Internet Network Information Center, Wind Database, China Insurance Industry Association website, National Finance and Development Laboratory, Financial Institution of Chinese Academy of Social Sciences, and the preliminary research results of the Investment and Financing Research Center of Chinese Academy of Social Sciences and the Zero-One Finance. Indicators such as population size, affluence, and agricultural economic level come from the China Statistical Yearbook. In the empirical test, natural logarithms are taken for all indicators. After taking the natural logarithm, the descriptive statistics of each indicator are shown in Table 1.

It can be seen from Table 1 that the standard deviation of agricultural NPS pollution levels is 1.159, which is relatively large, and the difference between the maximum (5.366) and the minimum (1.131) is significant. This indicates a significant difference in agricultural NPS pollution in various regions. The standard deviations of FinTech inclusive financial index, FinTech service use, FinTech service availability, FinTech infrastructure, and FinTech service quality are 0.183, 0.917, 0.859, 0.803, and 0.350, respectively. The gap between the maximum and minimum values of each indicator is relatively low. Hence, there is an insignificant difference in regional FinTech development levels. From a structural point of view, the variances of FinTech services use, the FinTech services availability and the FinTech service infrastructure are large. The standard deviation of population size and affluence are 0.935 and 0.297, respectively, which were relatively small. The standard deviation of the agricultural economic level is 1.155 and there is a large disparity between its maximum value (8.551) and its minimum value (4.732). As a matter of fact, the agricultural economic level has the largest standard deviation among all factors, and this factor may also be an important reason for the difference in the level of the agricultural NPS pollution. In this study, the agricultural economic level is selected as the threshold variable, because it is necessary and appropriate to reveal the different effects of FinTech and other explanatory variables on agricultural NPS pollution under different agricultural economic level intervals.

## 5. Results and Discussion

The impact of FinTech on agricultural NPS pollution is largely revealed from three aspects. The first aspect is to estimate the “STIRPAT” model and its improved model, and then to characterize the overall effect of FinTech on agricultural NPS pollution under these two models. The second part is to improve the “STIRPAT” model further. Instead of focusing on the impact of affluence and its squared term on agricultural NPS pollution, we pay more attention to the influence of FinTech and its squared term on agricultural NPS pollution. This is done to reveal and test whether the effect of FinTech development on agricultural NPS pollution is consistent with the characteristics of inverted “U” type relationship. Thirdly, regarding the agricultural economic level as the threshold variable, the threshold regression model is established to reveal the disparity of FinTech’s influence on agricultural pollution in different development stages of an agricultural economy. 

### 5.1. The Effect of FinTech under the “STIRPAT” Model

First, the “STIRPAT” model is estimated through a weighted least squares method. In order to compare the differential impact of FinTech development and its structure on agricultural NPS pollution, it is carried out from the perspective of the overall level and the structural level. The FinTech inclusive financial index is used to measure the FinTech development at the overall level; The structural level uses the FinTech service use index, the FinTech service availability index, the FinTech infrastructure index and the FinTech service quality index to measure the level of FinTech development. The estimated results are shown in model (1)–model (5) in Table 2. Later, the inspection process of this study also follows this operational idea, so it will not be described again. From the perspective of Adjusted R^2^, model (1)–model (5) have highly explanatory power. The heteroscedasticity test methods from Breusch-Pagan-Godfrey, Harvey, Glejser, ARCH, and White show that there is no heteroscedasticity problem in each model. Next, the effects of each variable are analyzed.

Overall, FinTech development has a negative impact on agricultural NPS pollution, which passes the significance test at 1%. This fully proves that the development of FinTech can restrain agricultural NPS pollution. At the structural level, the two variables of FinTech service use and FinTech service availability also have negative effects on agricultural NPS pollution and pass the test at the significance level of 1%, which is consistent with the overall test results. The impact of FinTech infrastructure on agricultural NPS pollution is significantly positive. There are two possible reasons: First, despite the rapid development of FinTech, the FinTech infrastructure needs to be further improved. Most places where FinTech infrastructure is relatively complete and sound are regions with a developed economy. This indicates that FinTech infrastructure needs to be further improved, the radiation area needs to be further expanded, and the contribution of green development needs to be further reflected. Finally, the impact of FinTech service quality on agricultural NPS pollution is insignificant. In summary, the influence of the overall FinTech development index and its structural index on agricultural NPS pollution is inconsistent.

From other variables, the effects of affluence on agricultural NPS pollution are significantly positive in model (1)–model (3) and model (5), consistent with theoretical analysis. However, it should be noted that in the model (4), the effect is markedly negative. On the whole, the impact of affluence on agricultural NPS pollution may be nonlinear, and there may be inverted “U” type features. Therefore, in the following, we will continue to use the improved “STIRPAT” model to test the inverted “U” type characteristics of the impact of affluence on agricultural NPS pollution. The impact of population size on agricultural NPS pollution is prominently positive and accordant in model (1)–model (5), consistent with theoretical analysis.

As the degree of affluence is quantified under different FinTech development indicators, the estimated effects are somewhat different. Next, we continue to establish the improved “STIRPAT” model to check the robustness of the impact of FinTech development and its structure on agricultural NPS pollution in this model. The estimated results from model (6)– model (10) are shown in Table 3. According to the results of Adjusted R^2^, each model has strong explanatory ability. It can be seen from the results of heteroscedasticity test that there is no heteroscedasticity in the estimation results of each model. Hence the estimation results are credible. What can be seen from each model is that the effects of the affluence and its second power on agricultural NPS pollution are significantly positive and negative, respectively. This indicates the relationship between agricultural NPS pollution and affluence conforms to the inverted “U” shape as in the Environmental Kuznets Curve, which is consistent with the conclusions of Dai et al. [37].

Next, we continue to observe the effect of FinTech development and its structure under the improved “STIRPAT” model. On the whole, the impact of FinTech development on agricultural NPS pollution is a markedly negative, which is consistent with the estimation of the former “STIRPAT” model without improvement. From a structural perspective, the FinTech service use index, FinTech service availability index and FinTech infrastructure index have a notably negative impact on agricultural NPS pollution. Moreover, FinTech service quality has a significantly positive impact on agricultural NPS pollution. Thus, the improved “STIRPAT” model works better than the unmodified model. It should be noted that FinTech service quality has a significant positive impact on agricultural NPS pollution in the improved model. This indicates that FinTech service quality needs to be further improved. 

### 5.2. Nonlinear Characteristics of the Impact of FinTech on Agricultural NPS Pollution

Compared with the improved “STIRPAT” model which focuses more on the nonlinear effects of affluence, this study is more interested in the nonlinear impact of FinTech development on agricultural NPS pollution. To this end, in this section, we introduce FinTech development and its second power into the model. The estimated results from model (11)–model (15) are shown in Table 4. According to Adjusted R^2^ and heteroscedasticity test, each model has strong explanatory ability and the estimation results are reliable.

**Figure 1:**
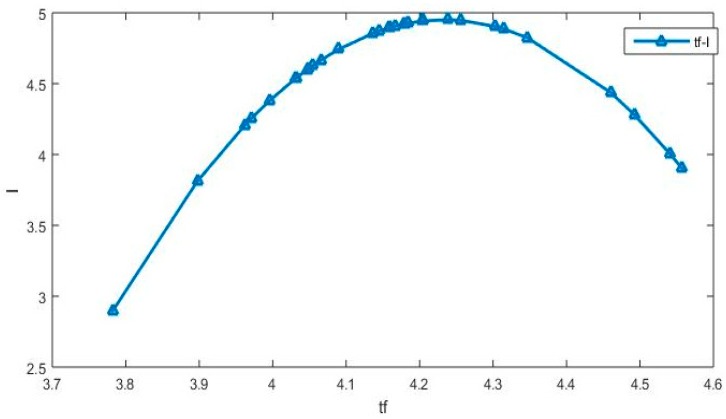
The impact of FinTech development on agricultural NPS pollution.

Overall, the effects of FinTech development and its second power on agricultural NPS pollution are significantly positive and negative, respectively. This shows that the impact of FinTech development on agricultural NPS pollution also exhibits an inverted “U” shape. In order to reflect this feature more intuitively, we draw the results of the impact of FinTech development on agricultural NPS pollution into Figure 1. It should be noted that when drawing Figure 1, we only focus on FinTech development and its secondary power, and other variables in “STIRPAT” model are placed in the random error term, which is not considered in the drawing. 

As can be seen from Figure 1, the initial stage of FinTech development is the improvement of financial service availability. Therefore, FinTech development will lead to scale expansion and increase the input of chemical fertilizers, pesticides, plastic mulch film and diesel oil. This in turn will increase pollutant emissions. Stated differently, financial development itself is a “double-edged sword” that can increase pollution emissions [47,48,49]. As an important component and innovative frontier of financial development, FinTech development is bound to result in this problem in the early days. This phenomenon is particularly evident in the agricultural sector that has long been excluded by financial institutions such as banks. However, with the further development of the integration of finance and technology, FinTech can play a role in curbing agricultural NPS pollution through technological progress, industrial restructuring, and human capital investment. In terms of technological progress, FinTech development can usher in technological progress, resolve the adverse selection and moral hazard issues in the field of agricultural financial services, reduce the energy consumption per unit of agricultural output, and curb agricultural NPS pollution. In terms of industrial structure, FinTech development can accelerate the development of “green finance”. This means more resources and funds will be flown into agricultural NPS pollution control. In terms of human capital investment, FinTech development can accelerate the improvement of farmers’ human capital and enhance their awareness of environmental protection.

From the structural perspective, the impact of FinTech service use and its second power on agricultural NPS pollution is different. The influence of FinTech service use on agricultural NPS pollution is significantly negative, but the effect of its second power is ambiguous. This shows that the impact of FinTech service use on agricultural NPS pollution is linear without an inverted “U” type relationship. The first and second power of FinTech service availability and FinTech infrastructure have significantly positive and negative effects on agricultural NPS pollution, respectively. This conforms to the inverted “U” type. The impact of the first and second power of FinTech service quality on agricultural NPS pollution are markedly positive and negative, respectively. This indicates that the effect of FinTech service quality on agricultural NPS pollution has a “U” type characteristic. That is to say, if the FinTech service quality is relatively low, agricultural NPS pollution will be more severe. Based on the FinTech service quality effect results under the above-mentioned “STIRPAT” model and its improved model, we can further conclude that we need to address the current dilemma of FinTech service quality. Furthermore, to enhance the role of FinTech in the control of agricultural NPS pollution, we must improve the FinTech service quality.

### 5.3. Threshold Effect of FinTech on Agricultural NPS Pollution

In the above section, we have revealed the impact of FinTech development on agricultural NPS pollution and its dynamic characteristics. Then, will this impact change with different development stages of an agricultural economy? This subsection aims to answer this question.

#### 5.3.1. Threshold Effect Test

The key to cross-section regression is to determine whether the threshold effect exists in the model and if yes, to determine the number of threshold values. According to the setting of the theoretical model, the existence of the threshold effect is tested first. Specifically, it is mainly carried out from the perspectives of the overall level and the structural level. At the overall level, the FinTech inclusive financial index is used to measure the level of FinTech development and reveal its impact on agricultural NPS pollution. At the structural level, the FinTech development level is measured from FinTech service use index, FinTech service availability index, FinTech infrastructure index and FinTech service quality index. Then the impacts of FinTech development structure on agricultural NPS pollution at different development stages of an agricultural economy are compared. To ensure robustness of the statistics, this study sets 1000, 2000, 3000, 4000, and 5000 bootstraps to simulate and calculate the values of the likelihood ratio statistic. The results from model (16)–model (20) are shown in Table 5.

As shown in Table 5, the LM values calculated by bootstrap simulation for 1000-5000 times are relatively robust at both the overall level and the structural level. Hence, 5000 simulation results are used as the basis for analysis. According to the results, at the overall level, the LM test value corresponding to the model is 12.053. From the corresponding p-value, it can be concluded that at the 5% significance level, the model rejects the null hypothesis of “no threshold effect”. At the structural level, the LM test values under the impact of FinTech service use, FinTech service availability, FinTech infrastructure and FinTech service quality on agricultural NPS pollution model are 12.044, 9.945, 13.054 and 12.26, respectively. It rejects the null hypothesis at the significance levels of 5%, 10%, 5%, and 5%, respectively. In summary, it is appropriate to select the threshold regression model for analysis.

**Figure 2:**
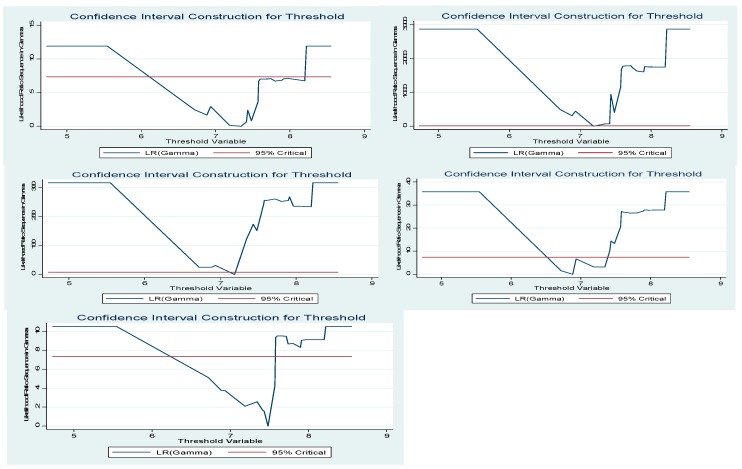
A significant test of the threshold effect.

From the perspective of the threshold value, considering the agricultural economic growth level as the threshold variable, the calculated thresholds of agricultural economic growth are 7.346, 7.189, 7.482, 6.885 and 7.188, respectively. Their corresponding confidence intervals are [6.718, 8.201], [7.189, 7.189], [6.718, 7.573], [6.718, 7.346], [7.189, 7.189], respectively. It can be seen from the results that 0 is not covered in the confidence interval, and the probability of “making a mistake” is only 5%. Therefore, under each model, the threshold of agricultural economic growth passes the test of significance. Moreover, the results in Table 5 are consistent with Figure 2. Thus, on the whole, whether at the overall level or the structural level, the model has significant threshold effects and thresholds, which can be further analyzed by threshold regression.

#### 5.3.2. Estimation of the Threshold Effect

Since this study uses the cross-section threshold regression model, there may be the problem of heteroscedasticity. To address heteroscedasticity, Hansen proposes two potential methods: one is to force the homoscedasticity hypothesis; and the other is to use the White test to correct the heteroscedasticity. These two methods can make regression results unbiased, effective and consistent. To be consistent with prior research, in this study, we use the White test. The estimated results from model (21)–model (25) are shown in Table 6, which show that the null hypothesis of “homoscedasticity” is accepted in all models. After correcting the heteroscedasticity by White test, the estimation results are more reliable. The joint R^2^ is greater than or close to 0.9, and each model has a good explanatory power.

At the overall level, when the agricultural economic growth level is in the range of *q* ≤ 7.346, the impact of FinTech development and its second power on agricultural NPS pollution is significantly positive and negative, respectively. This indicates an inverted “U” type curve with diminishing marginal effects. However, when the agricultural economic growth level is in the range of *q* > 7.346, the influence of FinTech development and its second power on agricultural NPS pollution are positive and negative, respectively, but not significant. This means that when an agricultural economy is at developing stage, FinTech will become a feasible way to control agricultural NPS pollution. When an agricultural economy is at developed stage, the suppression of agricultural NPS pollution by FinTech development becomes insignificant. This is because once the agricultural economy enters a developed stage, the agricultural industry becomes more advanced with high valued-added products and services. As a result, much more financial resources will be invested in agricultural industry.

As a result, FinTech’s advantages in terms of channel and efficiency can become insignificant, which will lead to its insignificant impact on agricultural NPS pollution. At the structural level, in the developing stage of an agricultural economy, the effects of the FinTech service use, the FinTech service availability, the FinTech infrastructure and their second power effects on agricultural NPS pollution are significantly positive and significantly negative, respectively. It indicates that at the structural level, the impact of FinTech development on agricultural NPS pollution also has an inverted “U” type characteristic. Moreover, FinTech service use, FinTech service availability and the maturity of FinTech infrastructure have a depressing effect on agricultural NPS pollution. Therefore, in the developing stage of an agricultural economy, it is more important to popularize the use of FinTech, promote the availability of farmers’ FinTech services and improve the FinTech infrastructure. In the developed stage of an agricultural economy, the impacts of FinTech service use, FinTech service availability, FinTech infrastructure and their second power on agricultural NPS pollution are still positive and negative, but not significant. Also, it should be noted that in the developing stage of an agricultural economy, the impact of FinTech service quality and its second power on agricultural NPS pollution is significantly positive and negative, respectively, showing a typical “U” type feature. In the developed stage of an agricultural economy, the impact of FinTech service quality and its second power on agricultural NPS pollution is not significant. As a consequence, in the developing stage of an agricultural economy, in addition to popularizing the use of FinTech, promoting the availability of farmers’ FinTech services, and improving the FinTech infrastructure, it is also necessary to improve FinTech service quality. Furthermore, at different development stages of an agricultural economy, the impact of affluence on agricultural NPS pollution is uncertain. The agricultural resource endowments vary widely. There are labor-saving agriculture like in the United States, capital-saving agriculture like in Japan, and neutral technology advanced agriculture like in Europe. Therefore, when farmers’ income increases, whether they follow the path of increasing agricultural output or realizing capital saving by increasing input for factors like pesticides and fertilizers need to be analyzed on a case-by-case basis. Finally, from model (16)–model (20), it can be seen that no matter in the developing or developed stage of an agricultural economy, population size has a significant positive impact on agricultural NPS pollution. This suggests that the impact of the agricultural population size on agricultural NPS pollution is relatively constant. Rural population density had the greatest contribution to agricultural NPS pollution [50]. Therefore, to achieve the goal of agricultural NPS pollution control, rural population size should be considered [51].

## 6. Conclusions

In this study, based on the theoretical models of IPAT and STIRPAT, we set up an econometric model to investigate the impact of FinTech development on agricultural NPS pollution. We estimate the model by weighted least square and cross-section threshold regression method. At the overall level as well as the structural level, we test the effects of FinTech on agricultural NPS pollution and its characteristics. In particular, we test how these effects depend on the different development stages of an agricultural economy. Our main findings include the following:

(1). Overall, the development of FinTech is a “double-edged sword”, and the effect of FinTech development on agricultural NPS pollution presents a typical inverted “U” type feature. That is, in the developing stage of an agricultural economy, the impact of FinTech development on agricultural NPS pollution is significantly positive. This will cause the agricultural production to expand in scale, resulting an aggravation in agricultural pollution emissions and an increase in agricultural NPS pollution. Only when the development of FinTech crosses the threshold value, its impact on agricultural NPS pollution will be significantly negative.
(2). At the structural level, the effects of FinTech service availability and FinTech infrastructure on agricultural NPS pollution also present an inverted “U” type relationship. This is consistent with the results at the overall level. However, the impacts of FinTech service use and FinTech service quality on agricultural NPS pollution are inconsistent with those obtained at overall level. Among them, the effect of FinTech service quality on agricultural NPS pollution has a “U” type relationship. Combined with the results from the overall level and the structural level, this indicates that achieving high-quality FinTech services will be the key for FinTech to support agricultural NPS pollution control in the future.
(3). From the perspective of the threshold effect, in the developing stage of an agricultural economy, the impact of FinTech development on agricultural NPS pollution also presents an inverted “U” type characteristic. In the developed stage of an agricultural economy, the influence of FinTech development and its second power on NPS pollution is insignificant. The influences of FinTech service use, FinTech service availability and FinTech infrastructure on agricultural NPS pollution also have an inverted “U” type features in developing stage of an agricultural economy. However, their effects become insignificant. Therefore, in the developing stage of an agricultural economy, it is even more important to popularize the use of FinTech, promote the availability of farmers’ FinTech services, and improve FinTech infrastructure to help control agricultural NPS pollution.

In summary, FinTech is the inevitable result of financial system evolution. It not only addresses the shortcomings of traditional financial system, but also brings new opportunities for the economic development and social governance, especially in developing countries. Agricultural NPS pollution is one of most important themes in front of the governments of developing countries. Whether we can control agricultural NPS pollution effectively will directly affect the objectives of traditional agriculture transformation and green development. Fortunately, FinTech has begun to play an important role in leading the green development of this field. Therefore, this paper contributes to the literature by linking FinTech with agricultural NPS pollution. There are many directions to be further studied in the future. First, we can investigate different FinTech applications in agricultural NPS pollution control and develop a comprehensive theoretical framework of the FinTech to support agricultural NPS pollution control. Second, we can design appropriate policies for agricultural NPS pollution control from the perspective of FinTech, especially for developing countries. Third, we can expand the control variables in agricultural NPS pollution from the perspective of behavior and system to further demonstrate the robustness of our results. 

## Figures and Tables

**Table 1:** Descriptive statistics for each variable.

Variable	Mean	Median	Maximum	Minimum	Standard Deviation
I	3.803	4.352	5.366	1.131	1.159
T_F_	4.168	4.159	4.557	3.782	0.183
T_U_	2.818	2.758	4.565	0.000	0.917
T_G_	2.881	2.907	4.542	0.536	0.859
T_I_	3.139	3.168	4.557	1.194	0.803
T_Q_	3.991	4.089	4.377	2.457	0.350
A	9.413	9.379	10.147	8.917	0.297
P	7.21	7.385	8.499	5.451	0.935
q	7.241	7.582	8.551	4.732	1.155

Note: The descriptive statistics of all variables are the results after the logarithm.

**Table 2:** “STIRPAT” model estimation results.

Variable		Model					
		FinTech Inclusive Financial Index (T_F_)	FinTech Service Use Index (T_U_)	FinTech Service Availability Index (T_G_)	FinTech Infrastructure Index (T_I_)	FinTech Service Quality Index (T_Q_)
		(1)	(2)	(3)	(4)	(5)
Constant		−6.51 (−15.53) **	−10.722 (−37.44) ***	−9.612 (−38.33) ***	−0.031 (−0.09)	−8.214 (−11.63) ***
T		−1.185 (−4.94) ***	−0.233 (−30.88) ***	−0.183 (−6.40) ***	0.256 (15.0) ***	−0.002 (−0.11)
A		0.732 (8.75) ***	0.676 (13.23) ***	0.561 (14.04) ***	0.544 (−11.33) ***	0.353 (4.87) ***
P		1.162 (53.34) ***	1.221 (53.26) ***	1.198 (112.93) ***	1.134 (103.04) ***	1.20 (31.34) ***
R^2^		0.999	0.999	0.999	0.998	0.976
Adjusted R^2^		0.999	0.999	0.999	0.997	0.973
F-statistic		13,561.21	22,573.21	54,332.30	4743.787	366.6
Heteroskedasticity Test	Breusch-Pagan-Godfrey	0.216	1.512	0.017	2.675	8.2
	Harvey	8.139	5.527	0.395	7.601	5.158
	Glejser	0.788	2.388	0.395	5.702	7.794
	ARCH	1.135	0.177	0.127	0.125	0.269
	White	0.470	50.187	7.831	1.202	34.41

Note: ** and *** indicate significant levels of significance at 1%, 5%, respectively. Non-marked is not significant.

**Table 3:** Estimation results of the improved STIRPAT model.

Variable		Model				
		FinTech Inclusive Financial Index (T_F_)	FinTech Service Use Index (T_U_)	FinTech Service Availability Index (T_G_)	FinTech Infrastructure Index (T_I_)	FinTech Service Quality Index (T_Q_)
		(6)	(7)	(8)	(9)	(10)
Constant		−134.25 (−9.89) ***	−207.745 (−9.17) ***	−177.496 (−5.92) ***	−302.183 (−11.16) ***	−167.82 (−9.98) ***
A		27.72 (9.73) ***	42.687 (8.813) ***	36.078 (5.62) ***	62.908 (11.02) ***	34.709 (9.76) ***
A^2^		−1.447 (−9.64) ***	−2.228 (−8.61) ***	−1.872 (−5.45) ***	−3.303 (−11.05) ***	−1.831 (−9.76) ***
T		−0.443 (−4.21) ***	−0.135 (−5.52) ***	−0.1 (−4.67) ***	−0.07 (−1.79) ***	0.042 (4.09) ***
P		1.026 (80.85) ***	1.066 (90.63) ***	1.103 (62.88) ***	0.965 (55.63) ***	1.0 (167.15) ***
R^2^		0.999	0.999	0.999	0.998	0.999
Adjusted R^2^		0.999	0.999	0.999	0.998	0.999
F-statistic		12,834.47	10,057.9	21,194.11	6239.518	24,790.43
Heteroskedasticity Test	Breusch-Pagan-Godfrey	0.564	1.673	0.589	0.530	3.464
	Harvey	3.349	4.063	2.56	2.686	8.722
	Glejser	1.245	2.978	0.625	1.214	2.775
	ARCH	0.448	3.601	0.007	0.65	2.704
	White	0.278	241.958	0.089	173.514	1.394

Note: *** indicates significant at the 1% significance level.

**Table 4:** Estimation of the impact of FinTech on NPS pollution.

Variable		Model				
		FinTech Inclusive Financial Index (T_F_)	FinTech Service Use Index (T_U_)	FinTech Service Availability Index (T_G_)	FinTech Infrastructure Index (T_I_)	FinTech Service Quality Index (T_Q_)
		(11)	(12)	(13)	(14)	(15)
Constant		−120.743 (−3.137) ***	−12.987 (−33.56) ***	−16.995 (−13.01) ***	−9.04 (−6.947) ***	−6.366 (−3.535) ***
T		53.238 (2.89) ***	−0.352 (−1.94) *	1.88 (5.15) ***	2.066 (7.13) ***	−0.781 (−1.96) *
T^2^		−6.519 (−2.997) ***	−0.01 (−0.247)	−0.405 (−6.613) ***	−0.342 (−7.542) ***	0.124 (2.057) **
A		0.943 (1.34) *	0.982 (21.83) ***	1.345 (9.59) ***	0.283 (2.29) **	0.314 (2.41) **
P		1.003 (9.223) ***	1.202 (81.20) ***	0.892 (54.42) ***	1.01 (45.79) ***	1.159 (36.71) ***
R^2^		0.851	0.999	0.998	0.993	0.998
Adjusted R^2^		0.827	0.999	0.998	0.992	0.998
F-statistic		35.775	3733930	5316.719	924.264	3851.973
Heteroskedasticity Test	Breusch-Pagan-Godfrey	1.34	0.591	0.127	22.421	0.594
	Harvey	1.845	7.909	7.780	11.673	24.838
	Glejser	1.663	0.741	0.361	12.490	1.493
	ARCH	1.329	2.212	0.033	0.297	1.407
	White	0.848	0.347	0.176	21.958	0.412

Note: ***, **, and * indicate significant levels of significance at 1%, 5%, and 10%, respectively.

**Table 5:** LM test based on Bootstrap.

Bootstrap	Model				
	FinTech Inclusive Financial Index (T_F_)	FinTech Service Use Index (T_U)_	FinTech Service Availability Index (T_G_)	FinTech Infrastructure Index (T_I_)	FinTech Service Quality Index (T_Q_)
	(16)	(17)	(18)	(19)	(20)
1000	12.053 (0.02)	12.043 (0.02)	9.945 (0.093)	13.054 (0.012)	12.26 (0.033)
2000	12.053 (0.022)	12.043 (0.024)	9.945 (0.113)	13.054 (0.016)	12.26 (0.027)
3000	12.053 (0.019)	12.044 (0.019)	9.945 (0.100)	13.054 (0.014)	12.26 (0.028)
4000	12.053 (0.018)	12.044 (0.021)	9.945 (0.09)	13.054 (0.015)	12.26 (0.024)
5000	12.053 (0.024)	12.044 (0.019)	9.945 (0.106)	13.054 (0.015)	12.26 (0.024)

Note: () is the *p*-value.

**Table 6:** Estimation results of the threshold model for the impact of FinTech on agricultural NPS pollution.

Variable	Model									
	FinTech Inclusive Financial (T_F_)		FinTech Service Use Index (T_U_)		FinTech Service Availability Index (T_G_)		FinTech Infrastructure Index (T_I_)		FinTech Service Quality Index (T_Q_)	
	(21)		(22)		(23)		(24)		(25)	
	*q* ≤ 7.346	*q* > 7.346	*q* ≤ 7.189	*q* > 7.189	*q* ≤ 7.482	*q* > 7.482	*q* ≤ 6.885	*q* > 6.885	*q* ≤ 7.188	*q* > 7.188
Constant	−143.787 ** [−233.18, −87.97]	−95.016 [−183.3, 13.65]	−17.8 [−37.95, 2.35]	−6.01 [−18.57, 6.55]	−25.882 ** [−76.66, −13.17]	−17.834 [−27.89, 3.86]	−53.723 ** [−81.41, −23.63]	−3.401 [−14.6, 6.96]	186.728 ** [27.81, 345.65]	−1.967 [−13.85, 9.91]
T	59.83 ** [ 30.38, 103.76]	43.265 [−13.01, 83.29]	0.854 ** [0.29,1.42]	0.20 [−1.45, 1.86]	1.684 ** [0.92, 2.40]	0.423 [−1.14, 4.3]	9.196 ** [0.71, 15.4]	0.927 [−0.326, 3.23]	−97.6 ** [−179.09, −16.11]	−0.1291 [−4.35, 4.09]
T^2^	−7.557 ** [−12.73, −4.16]	−5.162 [−9.89, 1.31]	−0.258 ** [−0.33, −0.18]	−0.057 [−0.28, 0.16]	−0.418 ** [−0.61, −0.31]	−0.146 [−0.75, 0.11]	−1.576 ** [−2.56, −0.24]	−0.144 [−0.52, 0.07]	12.413 ** [1.91, 22.92]	0.025 [−0.62, 0.67]
A	2.224 [−1.087, 4.39]	0.54 [−0.69, 2.62]	1.422 [−0.43, 3.27]	0.671 [−0.55,1.9]	2.06 ** [0.90, 7.01]	1.514 [−0.34, 2.48]	4.033 ** [1.97, 6.47]	0.205 [−1.04, 1.28]	0.143 [−0.54, 0.82]	0.3198 [−0.54, 1.18]
P	1.236 ** [0.30, 1.84]	0.495 ** [0.12, 1.31]	1.1 ** [0.45, 1.74]	0.528 ** [0.06, 1.00]	1.333 ** [0.79, 2.47]	1.031 ** [0.105, 1.40]	0.943 ** [0.69, 1.58]	0.581 ** [0.17, 0.96]	0.931 ** [0.715, 1.15]	0.458 ** [0.11, 0.81]
R^2^	0.915	0.377	0.898	0.315	0.943	0.613	0.9	0.474	0.833	0.284
Heteroskedasticity Test	0.336		0.389		0.587		0.338		0.892	
Joint R^2^	0.919		0.916		0.937		0.92		0.899	

Note: ** indicates significant at a 5% significance level, and no mark means not significant.
